# Childhood malignancy-associated hemophagocytic lymphohistiocytosis: a retrospective, single-center study of 44 patients

**DOI:** 10.3389/fimmu.2026.1801752

**Published:** 2026-05-07

**Authors:** Chengshuang Huang, Jia Huang, Qu Le, Yan Zhou

**Affiliations:** 1 Emergency Department, West China Second University Hospital, Sichuan University, Chengdu, China; 2 Key Laboratory of Birth Defects and Related Diseases of Women and Children, Ministry of Education, Sichuan University, Chengdu, China

**Keywords:** hemophagocytic lymphohistiocytosis, malignancy, overall response rate, overall survival, pediatric

## Abstract

**Purpose:**

This retrospective study aimed to investigate the clinical characteristics, management, and prognosis of pediatric malignancy-associated hemophagocytic lymphohistiocytosis (M-HLH).

**Methods:**

This was a retrospective, single-center cohort study, pediatric patients diagnosed with M-HLH diagnosed from January 2003 to December 2024 in our center were enrolled. Clinical characteristics, treatment regimens, overall response rate (ORR), and overall survival (OS) were evaluated. Univariate and multivariate analyses of potential factors were performed to identify prognostic factors.

**Results:**

Of the 44 patients, the median age at M-HLH diagnosis was 6.63 years (range: 0.33–15.58). Most patients (79.5%) developed HLH induced by tumors. Lymphoma was the most common malignancy (56.8%), predominantly of the T/NK - cell subtype (36.4%), followed by acute leukemia (27.3%) and Langerhans cell histiocytosis (15.9%). Marked elevations were observed in IL - 2R, IFN - γ, TNF - α, IL - 6, and IL - 10. Pathogenic variants in HLH - associated genes (*LYST*, *UNC13D*, and *XIAP)* were identified in three cases. Patients with the HLH at malignancy diagnosis were significantly older and had lower platelet and albumin levels compared to those with HLH after malignancy chemotherapy(*P* < 0.05). The ORR at the 4 week HLH diagnosis and at the final follow-up was 63.6% and 68.2%, respectively. The 6-month, 1-year, and 2-year OS rates were 79%, 69%, and 67%, respectively. Elevated serum ferritin (> 5000 ng/mL), age (> 10 years), lactate dehydrogenase (> 500 U/L), and failure to achieve CR were all associated with lower OS (*P* < 0.05). Achieving CR significantly enhanced OS in malignancy-induced HLH (*P* < 0.01), but not in chemotherapy-induced HLH. Failure to achieve CR by the final follow-up was an independent risk factor for a poor prognosis.

**Conclusions:**

The prognosis of pediatric M - HLH patients is poor. Remission of HLH is critical to the prognosis of M - HLH. Personalized and intensive treatment regimens are necessary for pediatric M - HLH.

## Introduction

Hemophagocytic lymphohistiocytosis (HLH) is a life-threatening but rare hyperinflammatory syndrome characterized by dysregulated proliferation and activation of immune cells (1). Its typical clinical manifestations include fever, hepatosplenomegaly, pancytopenia, hypertriglyceridemia, hypofibrinogenemia, and hyperferritinemia (2).HLH is broadly categorized into primary and secondary forms. Primary HLH, typically familial and driven by genetic defects, predominantly manifests in childhood. In contrast, secondary HLH (sHLH) arises in the absence of known genetic abnormalities and is frequently triggered by infections, autoimmune diseases, malignancies, or other factors (3).

Among sHLH subtypes, malignancy-associated HLH (M-HLH) has the highest mortality (4). The early clinical presentation of M-HLH is often complex and nonspecific, complicating timely diagnosis. Consequently, treatment strategies must be highly individualized and adapted to different disease stages (5). The prognosis of M-HLH remains poor, with reported 5-year OS rates (ranging from only 10% to 30% (6, 7). Notably, M-HLH occurs less frequently in the pediatric population than in adults, resulting in a paucity of large-scale studies specifically focused on children with this condition.

Given the distinct clinical context and the urgent need for early intervention in pediatric M-HLH, a comprehensive understanding of its unique features is essential. To address this gap, we conducted a retrospective analysis of clinical data from pediatric patients diagnosed with M-HLH at our institution over the past decade. This study aims to describe the clinical and laboratory characteristics of pediatric M-HLH and to identify potential risk factors associated with outcomes in this vulnerable population.

## Materials and methods

### Participants and treatment

**Figure 1:**
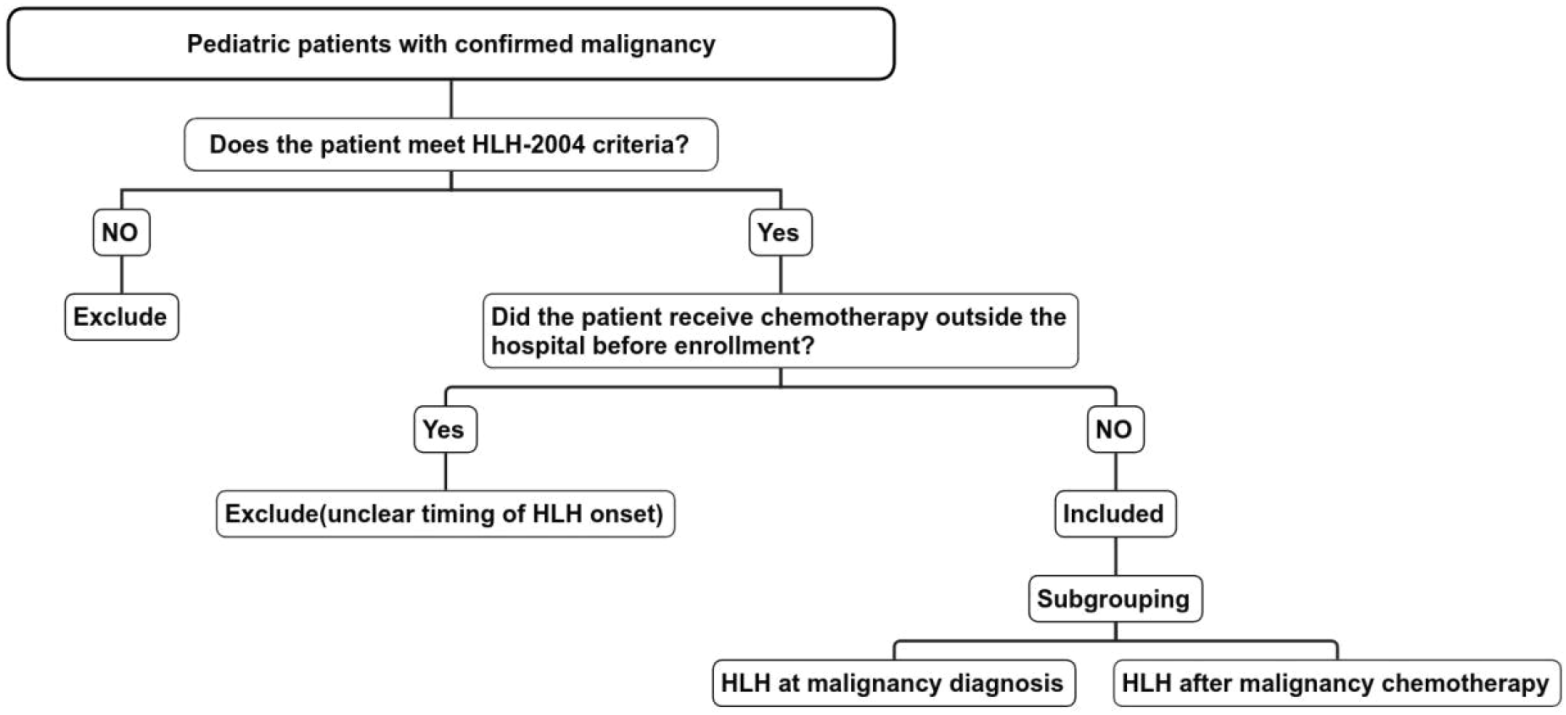
Flowchart of inclusion and exclusion criteria for M-HLH.

This retrospective cohort study included pediatric patients diagnosed with M-HLH at West China Second University Hospital, Sichuan University, between January 2003 and December 2024. The diagnosis of M-HLH was established based on the HLH-2004 diagnostic criteria (3), with confirmation of an underlying malignancy. Patients who had received any form of chemotherapy prior to admission were excluded from the analysis. The specific flowchart (**Figure 1**). In this study, Langerhans cell histiocytosis (LCH) is classified into single-system and multisystem types. In patients with multisystem involvement—particularly those with risk organ involvement—recurrence rates and mortality are elevated. In this study, all enrolled LCH cases were of the multisystem type with risk organ involvement; therefore, LCH was classified as a malignant tumor in this study.

HLH-directed induction therapy consisted of the HLH-1994 regimen or the HLH-2004 regimen, or monotherapy with glucocorticoids (3, 8). Treatment targeting the underlying malignancy was administered according to established protocols specific to each malignancy subtype.

Treatment response was assessed using the following criteria. Complete response (CR) was defined as the resolution of all HLH-related clinical signs and the normalization of all relevant laboratory parameters, including ferritin, soluble interleukin-2 receptor (sCD25), complete blood count, triglycerides, and alanine aminotransferase (ALT). Partial response (PR) was characterized by the attainment of normal body temperature, along with an improvement of ≥ 25% in at least two HLH-related symptoms or laboratory indicators. This included a reduction of sCD25 by at least one-third, a decrease in ferritin and triglyceride levels by ≥ 25%, a decline in ALT by ≥ 50% (if the baseline level was > 400 U/L), and a doubling of the complete blood count without transfusion support. No response (NR) was defined as failure to meet the criteria for either CR or PR. The overall response rate (ORR) was calculated as (CR + PR)/total number of evaluable patients × 100%. OS was defined as the interval from the diagnosis of HLH to death from any cause or the last follow-up. Patients were followed until December 2024. The study protocol was approved by the Ethics Committee of West China Second University Hospital and conducted in accordance with the principles of the Declaration of Helsinki.

### Statistical analysis

Statistical analyses were performed using SPSS software (version 22.0; IBM, Armonk, NY). Continuous variables with normal and skewed distributions were compared using the Student’s t-test and the Mann-Whitney U test, respectively. Categorical variables were compared using the Chi-square test or Fisher’s exact test, as appropriate. Survival analysis was performed using the Kaplan-Meier method, and differences between groups were compared with the log-rank test. Univariate and multivariate analyses of prognostic factors were conducted using Cox proportional hazards models. A two-sided P-value of < 0.05 was considered statistically significant.

## Results

### Baseline characteristics

A total of 44 pediatric patients with M - HLH were included in this study. The cohort consisted of 30 (68.2%) males and 14 (31.8%) females. The median age at HLH diagnosis was 6.63 years (range: 0.33–15.58).

Baseline clinical characteristics are summarized in **Table 1**. The majority of patients (35/44, 79.5%) was HLH at malignancy diagnosis. Lymphomas were the most common malignancy type (25/44, 56.8%), with T/NK - cell lymphoma being the predominant subtype (16/44, 36.4%), followed by acute leukemia (AL) (12/44, 27.3%) and LCH (7/44, 15.9%).

**Table 1:** Baseline M - HLH patient characteristics.

Characteristics	Median (range) or N (%)
Gender (male)	30 (68.2%)
Age ≥ 10 year	18 (40.9%)
Lymphoma subtype	
T/NK-cell lymphoma	16 (36.4%)
B-cell lymphoma	3 (6.8%)
Hodgkin lymphoma	3 (6.8%)
Unknown lymphoma type	3 (6.8%)
AL	
ALL	7 (15.9%)
MDS-AML	1 (2.3%)
JMML	4 (9.1%)
**LCH**	7 (15.9%)
The form of HLH	
The HLH at malignancy diagnosis	35 (79.5%)
The HLH after malignancy chemotherapy	9 (20.5%)
Clinical manifestations	
Fever	40 (91%)
Splenomegaly	32 (72.7%)
Hepatomegaly	37 (72.7%)
Lymphadenopathy	30 (68.2%)
Lab test	
EBV infection	13 (29.5%)
Neutrophil (×10^9^ /L)	1.09 (0.10 - 23.14)
Hemoglobin (g/L)	88.8 ± 17.8
Platelet (×10^9^ /L)	54 (8 - 289)
Ferritin (ng/mL)	1424.2 (106.30 - 40023)
Triglyceride (mmol/L)	2.23 (1 - 9.67)
Fibrinogen (g/L)	168 (50 - 669)
Aspartate aminotransferase (U/L)	90.5 (14 - 864)
Alanine aminotransferase (U/L)	53.5 (10 - 1750)
Lactate dehydrogenase (U/L)	773 (169- 2933)
Albumin (g/L)	32.8 ± 6.3
Total bilirubin (umol/L)	10.1 (2 - 102.6)
Activated Partial Thromboplastin Time (sec)	37.05 (19 - 507)
Prothrombin Time (sec)	12.8 (8.9 - 19.9)
Hemophagocytosis phenomenon in BM	30 (68.2%)

*ALL*, Acute Lymphoblastic Leukemia; *AML*, Acute Myeloid Leukemia; *MDS*, Myelodysplastic Syndrome; *LCH*, Langerhans Cell Histiocytosis; *JMML*, Juvenile Myelomonocytic Leukemia.

**Figure 2:**
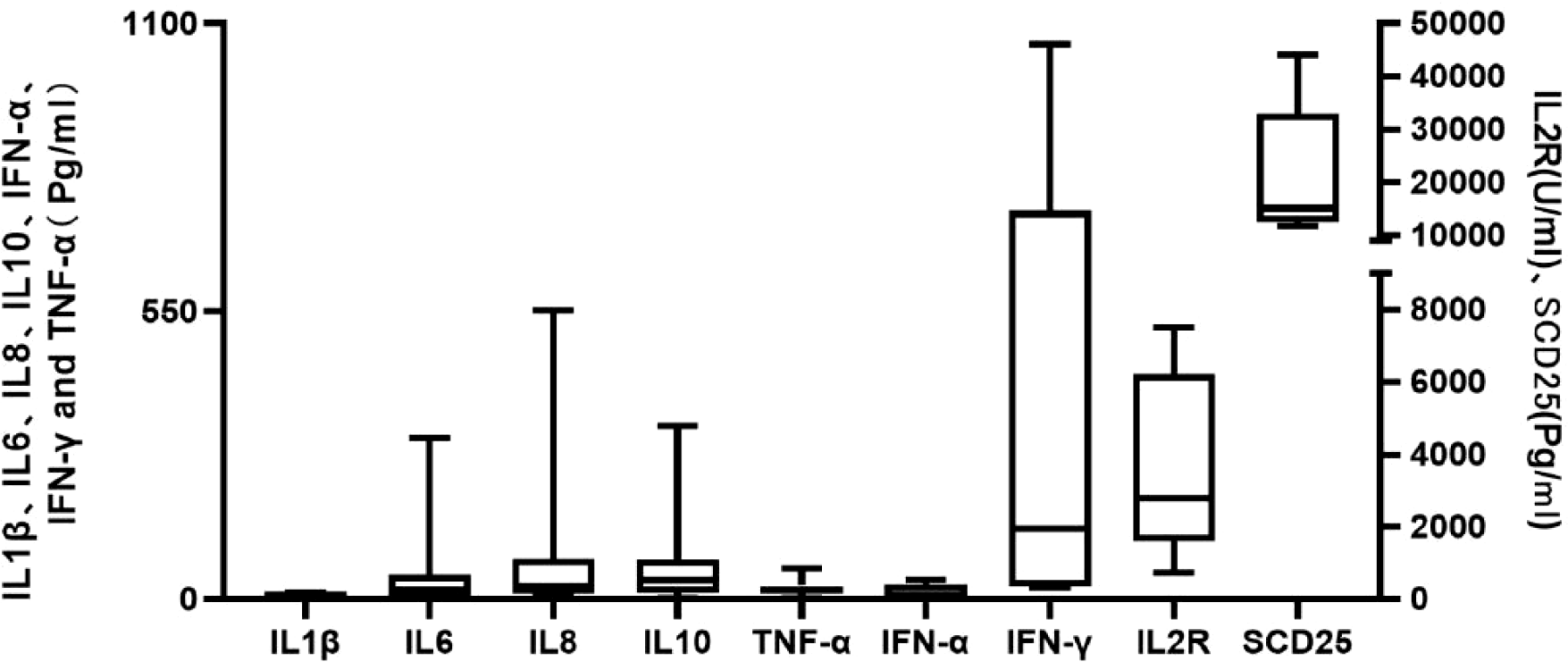
Laboratory results of cytokines and sCD25 in M-HLH.

Regarding clinical manifestations, fever was present in 40 (91.0%) patients. Hemophagocytosis was documented in bone marrow specimens of 30 (68.2%) patients, and 13 (29.5%) patients had evidence of EBV infection. Laboratory findings revealed that SF levels exceeded 500 ng/mL in 41 patients, with five patients having levels >10, 000 ng/mL. Cytopenia involving at least one lineage was observed in 40 patients, with 34 exhibiting bi- or tricytopenia. Among the eight patients tested for NK-cell activity and sCD25, four had low or absent NK-cell activity, and seven had elevated sCD25 levels (**Figure 2**). Cytokine testing was performed in 17 patients, including IL−1β, IL−2R, IL−6, IL−8, IL−10, TNF−α, IFN−α, and IFN−γ. Marked elevations were observed in IL−2R, IFN−γ, TNF−α, IL−6, and IL−10 (**Figure 2**).

HLH genetic testing was performed in 10 patients, and pathogenic variants in HLH−associated genes (*LYST*, *UNC13D*, and *XIAP*) were identified in three cases. The details are as follows: one patient with LCH carried an exon 2 deletion mutation in the *XIAP* gene and developed HLH during chemotherapy, with EBV negativity; one patient with B−cell lymphoma carried missense mutations in exon 19 of the *UNC13D* gene and exon 6 of the *LYST* gene, with HLH occurring at the time of malignancy diagnosis and EBV positivity; and one patient with T−cell lymphoma carried missense mutations in exon 27 of the *UNC13D* gene, as well as in exons 20 and 31, also presenting with HLH at malignancy diagnosis and EBV positivity.

A comparative analysis of patients with HLH at malignancy diagnosis showed that those with HLH after malignancy chemotherapy were significantly older (*P* = 0.008) and had significantly lower platelet counts and albumin levels (*P* = 0.050 and *P* = 0.030, respectively) (**Table 2**).

**Table 2:** Baseline characteristics of patients with Malignancy-induced HLH and Chemotherapy-induced HLH.

Characteristics	HLH at malignancy diagnosis group	HLH after malignancy chemotherapy group	*P* value
Age (years)	9.83(0.33-15.58)	2.33(0.33-12.75)	0.008
Gender(Male/Female)	23	7	1
Fever (Yes)	31	9	1
Lymphadenectasis	27	3	0.06
Hepatomegaly	29	8	0.65
Splenomegaly	26	6	0.42
Hemophagocytosis phenomenon in BM	22	8	0.46
EBV infection	12	1	0.24
Neutrophil (×10^9^ /L)	2.50 ± 4.80	3.97 ± 5.98	0.425
Hemoglobin (g/L)	87.91 ± 18.84	92.00 ± 14.31	0.53
Platelet (×10^9^ /L)	69.68 ± 55.71	114.20 ± 78.96	0.05
Ferritin (ng/ml)	4778.94 ± 7747.37	1722.45 ± 1701.30	0.226
Triglyceride (mmol/L)	2.73 ± 1.56	2.09 ± 0.96	0.225
Fibrinogen (g/L)	205.44 ± 132.72	270.50 ± 126.75	0.176
Aspartate aminotransferase (U/L)	195.88 ± 199.44	123.70 ± 221.43	0.332
Alanine aminotransferase (U/L)	190.32 ± 332.62	104.40 ± 146.80	0.434
Lactate dehydrogenase (U/L)	1168.79 ± 794.87	693.70 ± 687.51	0.095
Albumin (g/L)	31.70 ± 5.82	36.57 ± 6.65	0.03
Total bilirubin (umol/L)	19.11 ± 24.16	16.58 ± 9.36	0.749
Activated Partial Thromboplastin Time (sec)	56.72 ± 82.47	35.45 ± 10.70	0.424
Prothrombin Time (sec)	13.13 ± 1.98	12.05 ± 2.73	0.171

Analysis across malignancy subtypes (lymphoma, AL, and LCH) showed significant differences in age distribution, with the LCH group being the oldest and the AL group the youngest (*P* < 0.001). Lymphadenopathy occurred most frequently in the lymphoma group (*P* = 0.030). Furthermore, Lymphoma-associated HLH mainly occurs at the time of malignancy diagnosis., whereas LCH-HLH primarily occurs after chemotherapy for malignancy (*P* < 0.001). No other laboratory parameters differed significantly among groups (**Supplementary Table 1**).

### Treatment and response

Among the 44 patients, four (9.1%) received no HLH-directed induction therapy. Induction regimens included glucocorticoid monotherapy in 20 (45.5%) patients, combined glucocorticoid and etoposide in 10 (22.7%) patients, and the HLH-94 or HLH-2004 protocol in 10 (22.7%) patients (median treatment duration: 1–10 months). For the underlying malignancy, four (9.1%) patients received no specific direct treatment for the malignancies, 31 (70.5%) received chemotherapy alone, and nine (20.5%) underwent chemotherapy combined with hematopoietic stem cell transplantation (HSCT).

**Figure 3:**
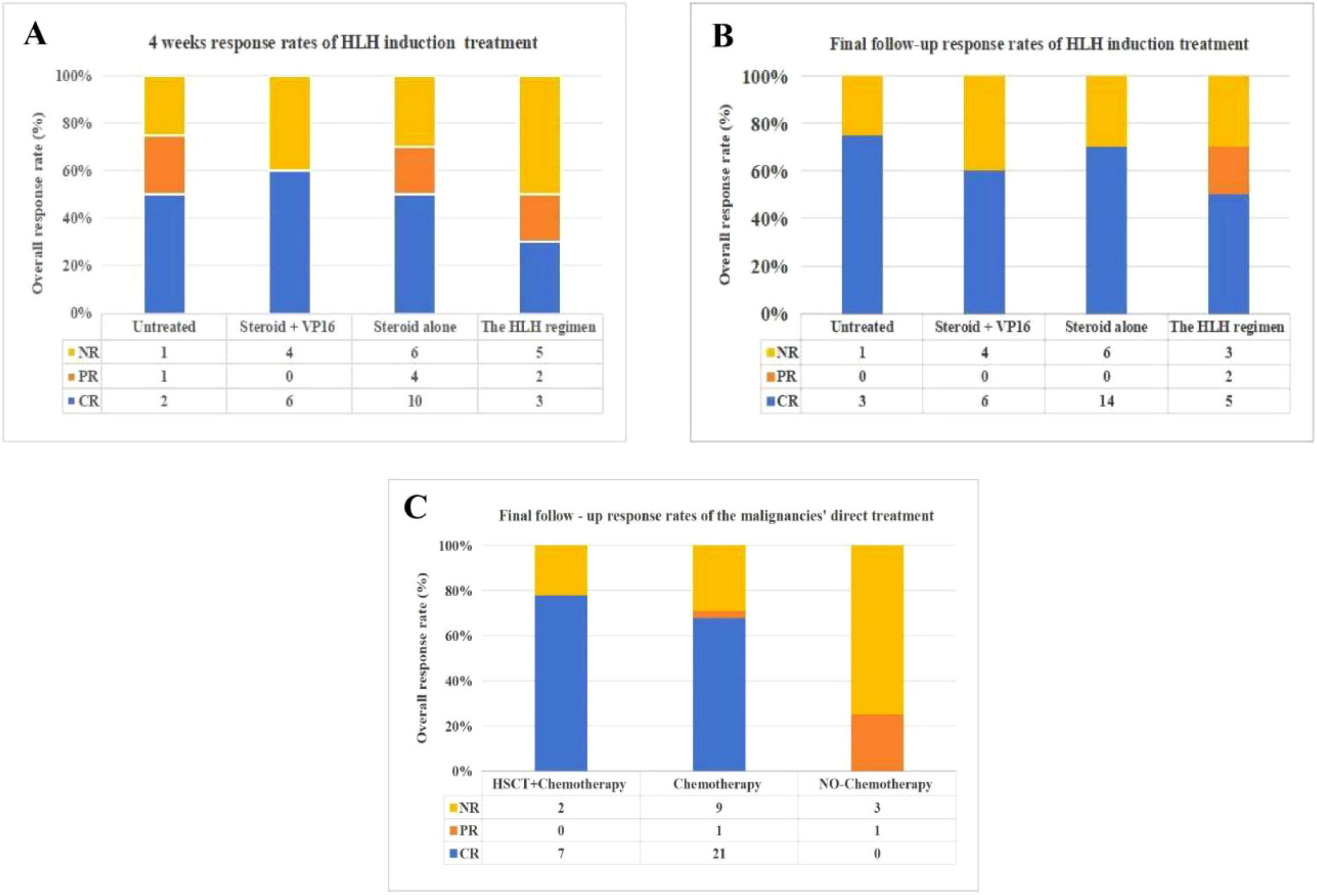
The response rates for HLH induction and the malignancies' direct treatment.Three grouped bar charts labeled **(A)**, **(B)** and **(C)** compare overall response rates for different HLH treatment regimens and malignancy treatments over time. Chart **(A)** shows 4-week response rates for HLH induction treatments, Chart **(B)** depicts final follow-up response rates for the same treatments, and Chart **(C)** presents final follow-up response rates for malignancy treatments. Each bar includes proportions for CR (complete response, blue), PR (partial response, orange), and NR (no response, yellow), with corresponding numeric values below each regimen for clarity.

Treatment response was evaluated at 4 weeks after HLH diagnosis and at the final follow-up. At the 4-week assessment, the ORR was 63.6% (95% CI: 48.6%–78.0%), with CR in 21 (47.7%) patients, PR in seven (15.9%) patients, and NR in 16 (36.4%) patients. By the final follow-up, the ORR increased to 68.2% (95% CI: 53.4%–83.0%). with 28 (63.6%) patients achieving CR, two (4.5%) in PR, and 14 (31.8%) with NR (**Figure 3**).The ORR at both time points did not differ significantly among the different HLH induction regimens (*P* = 0.73 and *P* = 0.961, respectively). This lack of difference persisted in subgroup analyses of both the HLH at malignancy diagnosis and HLH after malignancy chemotherapy groups (*P* > 0.05).

Given that HSCT is rarely performed within 4 weeks of HLH diagnosis, its impact was assessed only on the final ORR. Among 40 evaluable patients who received antitumor therapy, no significant difference in final ORR was observed between those treated with chemotherapy plus HSCT versus chemotherapy alone (*P* = 1.000). Subgroup analyses in the HLH at malignancy diagnosis and HLH after malignancy chemotherapy groups similarly showed no significant effect of treatment regimen on final ORR (*P* > 0.05).

### Survival outcome

**Figure 4:**
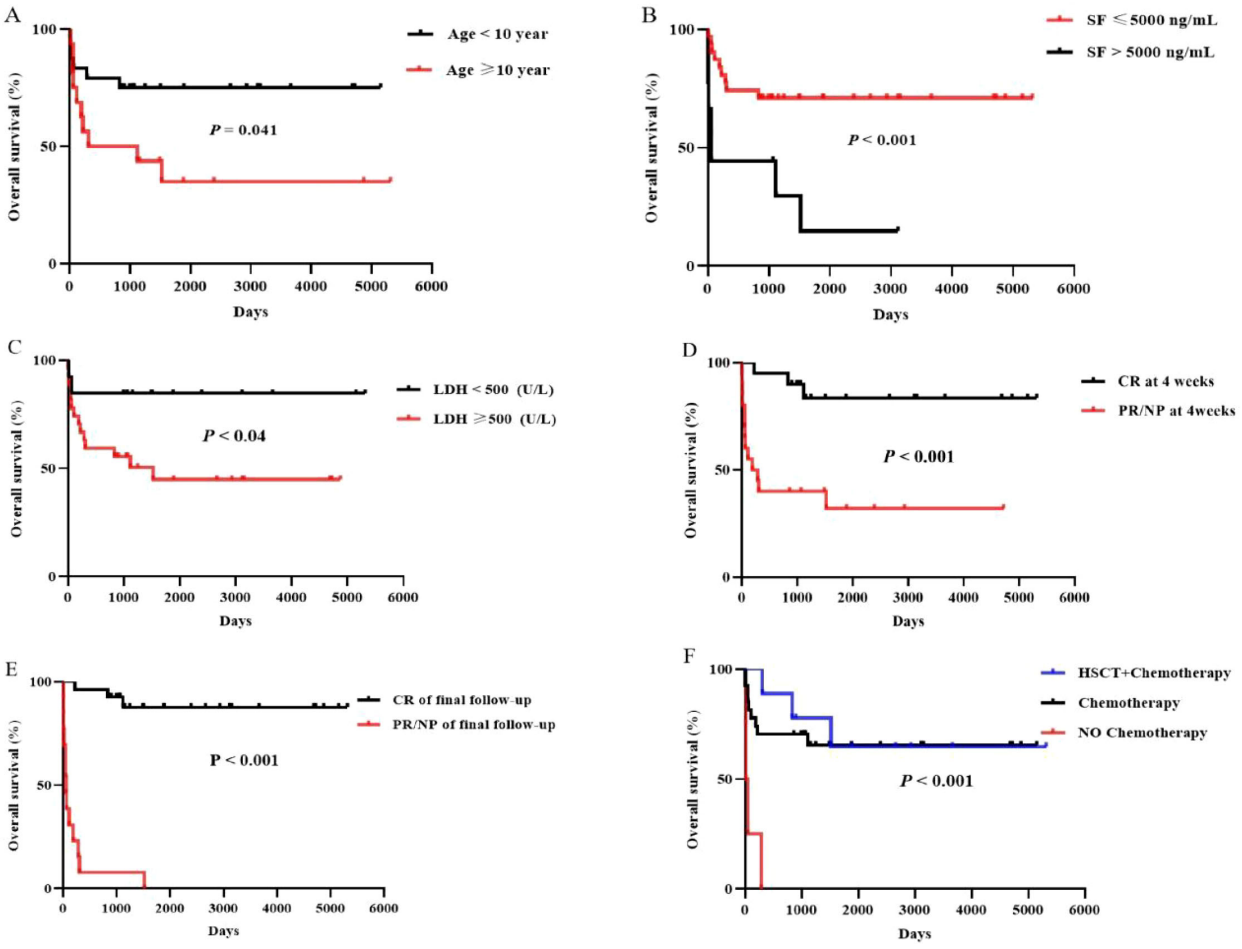
Overall survival of patients with M - HLH according to different influencing factors.– Panel of six Kaplan-Meier survival curve graphs illustrating overall survival percentages across various days for different clinical factors in a cohort. Panel **(A)** compares age under and over ten years; younger patients show higher survival. Panel **(B)** shows patients with serum ferritin below or above five thousand nanograms per milliliter; lower ferritin correlates with improved survival. Panel **(C)** compares lactate dehydrogenase below or above five hundred units per liter, with lower levels indicating better prognosis. Panel **(D)** distinguishes complete response versus partial or no response at four weeks; complete response associates with higher survival. Panel **(E)** contrasts complete response versus partial or no response at final follow-up, favoring complete response. Panel **(F)** compares hematopoietic stem cell transplantation plus chemotherapy, chemotherapy alone, and no chemotherapy, with combination therapy showing the best outcomes. Each panel provides log-rank P values indicating statistical significance.

With a median follow - up of 305 days (range: 15–1268), loss to follow - up occurred in 4 patients (9.1%).The median OS was not reached for the entire cohort. The 6 - month, 1 - year, and 2 - year OS rates were 79.0% (95% CI 64.2%–88.2%)、69.0% (95% CI 53.0%–80.5%) and 67.0% (95% CI 51.3%–78.7%), respectively. Survival analysis revealed that the type of HLH induction therapy, malignancy subtype, EBV infection status, and the form of HLH did not significantly affect OS (*P* = 0.904, *P* = 0.507, *P* = 0.949, and *P* = 0.360, respectively). Failure to receive any antitumor therapy was associated with significantly worse OS (*P* < 0.001) (**Figure 4**). However, no additional survival benefit was observed with the combination of HSCT and chemotherapy compared to chemotherapy alone (*P* = 0.817).

**Figure 5:**
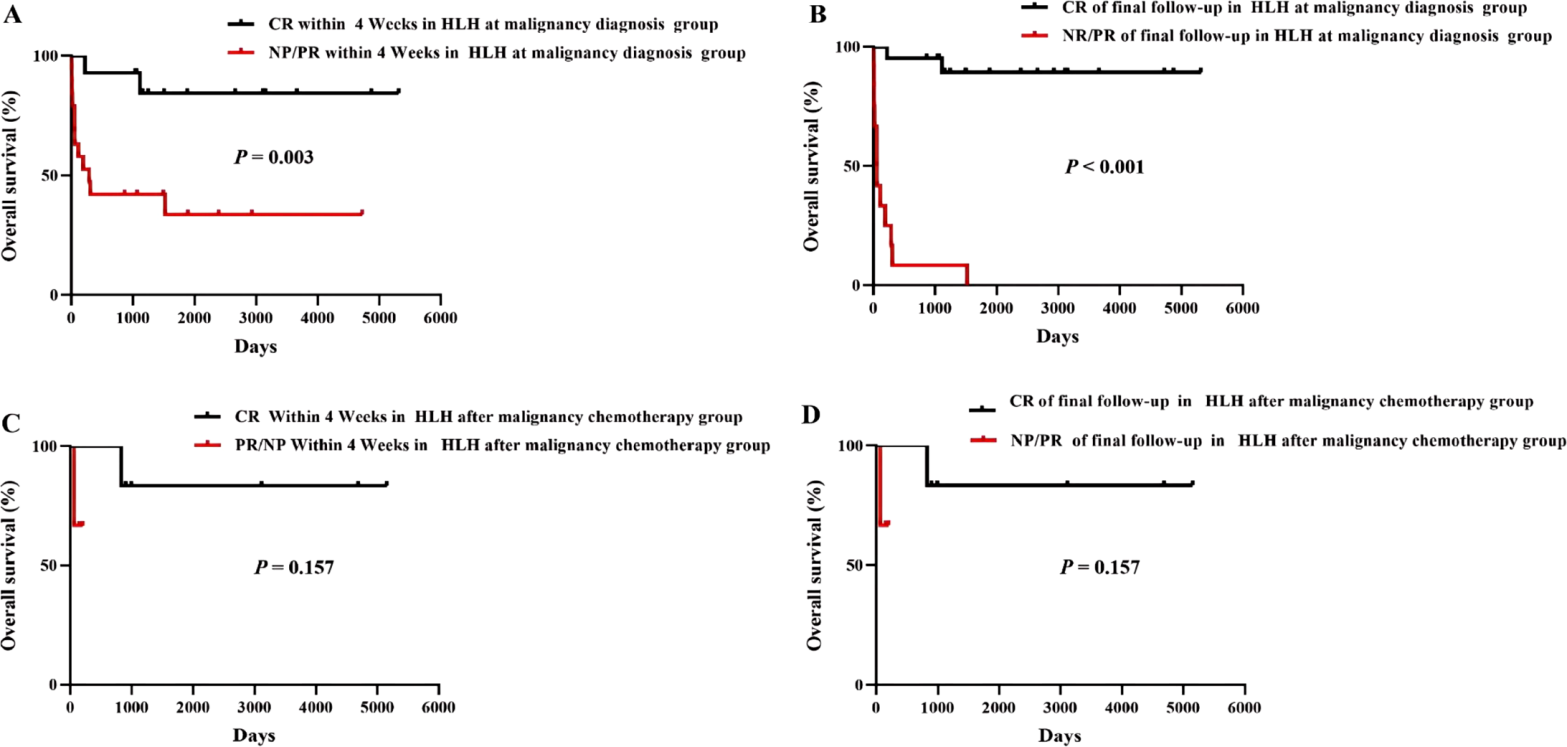
Overall survival of patients with CR of HLH in the HLH at malignancy diagnosis group and the HLH after malignancy chemotherapy group.Four-panel Kaplan-Meier survival analysis displaying overall survival percentages over days for different response groups in HLH patients, with panels **(A)** and **(B)** showing significant survival differences (P = 0.003 and P < 0.001) and panels **(C)** and **(D)** showing no significant differences (P = 0.157).

Several baseline factors predicted inferior OS: serum ferritin >5000 ng/mL (*P* < 0.001), age >10 years (*P* = 0.041), and lactate dehydrogenase >500 U/L (*P* = 0.040). Achieving CR of HLH at both the 4 - week assessment and final follow - up was strongly associated with improved OS compared to patients with PR or NR (*P* < 0.001) (**Figure 4**). Notably, in the HLH after malignancy chemotherapy group, achieving CR at either timepoint did not significantly influence OS (*P* = 0.157). In contrast, in the HLH at malignancy diagnosis group, CR achievement at both 4 weeks and final follow - up was significantly associated with better survival (*P* = 0.003 and *P* < 0.001, respectively) (**Figure 5**).

### Multivariate analysis of risk factors for mortality in M-HLH

Univariate analysis indicated that age <10 years, serum ferritin ≤5000 ng/mL, achieving CR at 4 weeks, and achieving CR at the final follow-up were significantly associated with improved survival (*P* = 0.003, *P* = 0.050, *P* < 0.001, and *P* = 0.003, respectively). A platelet count ≥100×10^9^/L, aspartate aminotransferase < 80 U/L, lactate dehydrogenase <500 U/L, and receipt of anti-tumor therapy showed a trend toward better survival but did not reach statistical significance (*P* = 0.086, *P* = 0.075, *P* = 0.059, and *P* = 0.080, respectively).

In the multivariate Cox regression model incorporating significant univariate factors, achieving CR at the final follow-up remained an independent protective factor for OS(*P* < 0.001; **Table 3**).

**Table 3:** Factor analysis of risk factors for death in M-HLH.

Factors	Univariate analysis			Multivariate analysis		
	HR	95%CI	P value	HR	95%CI	P value
Age < 10 years	0.363	0.131-1.001	0.05			
PLT ≥ 100×10^9^/L	0.17	0.022-1.286	0.086			
SF ≤ 5000 ng/mL	0.219	0.081-0.59	0.003			
AST < 80 U/L	0.358	0.115-1.111	0.075			
LDH < 500 U/L	0.239	0.054-1.054	0.059			
The malignancies' direct treatment	0.506	0.237-1.084	0.08			
CR at the final follow-up	0.036	0.01-0.138	< 0.001	0.036	0.01-0.138	<0.001
CR at 4 weeks	0.149	0.042-0.529	0.003			

*AST, Aspartate aminotransferase; LDH*, Lactate dehydrogenase; *SF*, Serum ferritin; *PLT*, Platelet count.

## Discussion and conclusion

HLH is a hyperinflammatory syndrome with a multifactorial etiology, encompassing genetic mutations, infections, malignancies, immunodeficiencies, and other triggers (9). M-HLH represents a common subtype in adults, accounting for 30–50% of secondary HLH cases (5). In contrast, its incidence is notably lower in the pediatric population, constituting only approximately 8% of childhood HLH cases (9). This study retrospectively analyzed 44 pediatric patients with M-HLH to delineate their clinical and laboratory characteristics and to identify prognostic risk factors.

The clinical phenotype of pediatric M-HLH in our cohort was characterized by persistent fever, hepatosplenomegaly, cytopenias, and bone marrow hemophagocytosis. EBV co-infection was detected in 29.5% of patients. Lymphoma was the most common underlying malignancy, suggesting that the oncologic process itself is a primary driver of HLH pathogenesis in these cases. These features are largely consistent with previous reports on pediatric M-HLH (10–13). However, notable epidemiological differences exist across studies. Reported median ages at diagnosis vary from 3.6 to 12 years (11, 14, 15). Furthermore, while some studies identify AL as the most frequent malignancy preceding HLH and highlight chemotherapy as a major trigger (13–16), our data revealed a distinct pattern: lymphoma was predominant, and chemotherapy-related HLH occurred most frequently in patients with LCH, followed by those with leukemia. We also observed significant heterogeneity in age distribution across malignancy subtypes, with the LCH subgroup being the oldest and the leukemia cohort the youngest. Importantly, patients who developed HLH prior to chemotherapy were significantly older and had lower platelet counts and albumin levels than those with post-chemotherapy HLH. This clinical variability underscores the need for future multicenter studies to establish standardized diagnostic approaches and risk-stratification models for pediatric M-HLH.

In this study, we identified genetic variants in *LYST*, *UNC13D*, and *XIAP* among patients with M-HLH. The patient carrying the *XIAP* variant developed HLH after tumor chemotherapy and was EBV-negative, whereas the patients with *LYST* and *UNC13D* variants had HLH occurring at the time of tumor diagnosis and were EBV-positive. These genes are known to cause degranulation dysfunction in cytotoxic lymphocytes, representing a core immunodeficiency underlying HLH (3). The synergistic interplay among genetic susceptibility, malignancy, chemotherapy, and infection has also been reported in previous studies. One case involved a patient with peripheral T−cell lymphoma who carried a heterozygous germline mutation in an HLH−associated gene, while the parents remained unaffected; the malignancy acted as a “second hit” triggering HLH, and the patient exhibited degranulation dysfunction of NK/T cells, with both the tumor and HLH achieving remission after transplantation (17).Another case described a patient with Ewing sarcoma and EBV co-infection, in which tumor burden and EBV activation together contributed to the development of HLH and multiple organ failure (18). Additionally, a patient with acute monocytic leukemia harboring FLT3-ITD/DNMT3A mutations developed typical HLH during the myelosuppressive phase following chemotherapy, suggesting that chemotherapy can trigger an immune storm in the context of genetic susceptibility (19). In summary, the pathogenesis of M-HLH results from the synergistic interaction of genetic predisposition with multiple factors, including malignancy, chemotherapy, or EBV infection, ultimately driving uncontrolled immune activation.

HLH is driven by excessively activated T cells and macrophages, which secrete a broad range of proinflammatory cytokines (20, 21). In this study, children with M-HLH exhibited markedly elevated levels of IL-10, IL-6, TNF-α, IFN-γ, IL-2R, and sCD25, consistent with the cytokine profile reported in adult M-HLH (21).This finding also suggests that the patterns of cytokine activation in pediatric and adult M-HLH are similar. Previous studies have identified high IL-2R levels as a poor prognostic factor in adult HLH (22). sCD25, a marker of T−cell activation, reflects the degree of inflammatory activity in HLH (23).IL-2R is a trimeric (α/β/γ) protein on the lymphocyte surface, and its α chain (CD25) is shed into the circulation to form sCD25. Since sCD25 testing is not routinely available in most hospitals, whether IL-2R can be used as an alternative to sCD25 to assess the activation status of HLH in clinical practice warrants further investigation. Due to the limitations of the retrospective study design and sample size, only two children with HLH occurring after tumor chemotherapy underwent cytokine testing in this study, both of whom presented with a marked cytokine storm.

The management of M-HLH centers on two pillars: control of the hyperinflammatory state and treatment of the underlying malignancy (5). HLH-directed therapies, particularly the HLH-94/04 protocols, remain the standard first-line induction regimens. Corticosteroids effectively modulate the cytokine storm (24), and early etoposide-based chemotherapy has demonstrated survival benefits across HLH subtypes (25, 26), though its cumulative toxicity and potential synergistic adverse effects in patients with malignancies warrant careful consideration (27, 28). In our cohort, different HLH induction regimens did not yield significant differences in ORR or OS. This finding supports a tailored treatment approach, where the choice and intensity of HLH-directed therapy should be guided by individual assessment of disease severity rather than a rigid protocol (24, 29). Ultimately, controlling the underlying malignancy is paramount, and cytotoxic chemotherapy forms the cornerstone of this effort (4). Our data confirm its critical therapeutic role. For patients with primary or refractory/relapsed disease, HSCT is often the only curative option (30). Interestingly, in our study, the combination of chemotherapy and HSCT did not confer a significant survival advantage over chemotherapy alone. This may be attributed to the histopathological diversity of the underlying malignancies or selection bias. These results collectively emphasize the necessity of individualized therapeutic stratification in pediatric M-HLH.

In our study, the ORR was 63.6% at 4 weeks post-diagnosis and improved to 68.2% at the final follow-up, rates consistent with prior pediatric M-HLH cohorts (15). The impact of HLH on the prognosis of the underlying malignancy is profound. Previous studies have shown that HLH is independently associated with induction failure and increased mortality in acute myeloid leukemia (31), and that pediatric patients undergoing HSCT who achieved resolution of HLH significantly improves survival outcomes (32). Our findings corroborate that achieving CR of HLH is strongly associated with improved survival. A critical subgroup analysis, however, revealed a key nuance: in patients who developed HLH after chemotherapy initiation, achieving CR did not translate into a significant survival benefit. In contrast, for patients with HLH onset *prior* to chemotherapy, CR was a powerful predictor of better outcomes. This differential effect suggests that the therapeutic algorithm and priority stratification may need to be adjusted based on the temporal relationship between HLH onset and chemotherapy.

The survival pattern observed in our cohort—with 6-month, 1-year, and 2-year OS rates of 79%, 69%, and 67%, respectively—aligns with previous reports (1, 10, 14). This pattern likely reflects improved early survival due to heightened clinical awareness and prompt HLH-directed intervention, while long-term prognosis remains heavily influenced by the control of the underlying malignancy and associated comorbidities (33). Identifying reliable prognostic biomarkers is therefore crucial for risk stratification. In adult M-HLH, extremely high serum ferritin (>15, 000 µg/L) and sCD25 (>10, 000 U/mL) are established poor prognostic markers (34, 35) In pediatric series, hypoalbuminemia, severe anemia, and bone marrow hemophagocytosis at diagnosis have been linked to worse outcomes (10, 15). Our study contributes to this prognostic framework by identifying serum ferritin >5000 ng/mL, age >10 years, and elevated lactate dehydrogenase (> 500 U/L) as significant predictors of inferior OS in pediatric M-HLH.

## Limitations and future directions

This study has several limitations. First, as a retrospective, single-center study, its findings may have limited generalizability and potential selection bias. Data completeness depended on medical records; key variables were consistently recorded, but certain immunological parameters and genetic testing results were incomplete, limiting their inclusion in prognostic analyses. Second, the small sample size (n=44) precluded robust multivariate analyses to adjust for confounders such as malignancy subtype, timing of HLH onset, and treatment intensity; thus, the identified prognostic factors should be considered exploratory. Third, heterogeneity in underlying malignancies and treatment protocols introduced unmeasured confounding, and subgroup analyses were underpowered due to limited sample sizes. Fourth, variability in follow−up duration may have introduced information bias regarding long−term outcomes, despite telephone follow−up for missing survival data. Nonetheless, this study provides valuable real−world data on pediatric M−HLH and highlights key features warranting further investigation in prospective, multicenter studies.

## Data Availability

The data analyzed in this study is subject to the following licenses/restrictions: In accordance with ethical principles and to promote research reproducibility, de-identified data supporting the findings of this study are available upon reasonable request to qualified researchers. All requests must be submitted in writing to the corresponding author and will require a formal data use agreement and approval from the aforementioned ethics committee. Requests to access these datasets should be directed to Yan Zhou, zhouyan810@scu.edu.cn.
