# Supplementary material for: Childhood malignancy-associated hemophagocytic lymphohistiocytosis: a retrospective, single-center study of 44 patients
Source: Front Immunol. 2026 May 7;17:1801752. doi: 10.3389/fimmu.2026.1801752 (PMC13189721; doi:10.3389/fimmu.2026.1801752)
Supplement: Supplementary file 1 [file DataSheet1.zip › SupMaterial/Table 2.docx]

Table 2 Baseline characteristics of patients with Malignancy-induced HLH and Chemotherapy-induced HLH

|  | **Malignancy-induced HLH group** | **Chemotherapy-induced HLH group** | ***P* value** |
| --- | --- | --- | --- |
| Age (years) | 9.83(0.33-15.58) | 2.33(0.33-12.75) | 0.008 |
| Gender(Male/Female) | 23 | 7 | 1 |
| Fever (Yes) | 31 | 9 | 1 |
| Lymphadenectasis | 27 | 3 | 0.06 |
| Hepatomegaly | 29 | 8 | 0.65 |
| Splenomegaly | 26 | 6 | 0.42 |
| Hemophagocytosis phenomenon in BM | 22 | 8 | 0.46 |
| EBV infection | 12 | 1 | 0.24 |
| Neutrophil (×10^9^ /L) | 2.50 ± 4.80 | 3.97 ± 5.98 | 0.425 |
| Hemoglobin (g/L) | 87.91 ± 18.84 | 92.00 ± 14.31 | 0.53 |
| Platelet (×10^9^ /L) | 69.68 ± 55.71 | 114.20 ± 78.96 | 0.05 |
| Ferritin (ng/ml) | 4778.94 ± 7747.37 | 1722.45 ± 1701.30 | 0.226 |
| Triglyceride (mmol/L) | 2.73 ± 1.56 | 2.09 ± 0.96 | 0.225 |
| Fibrinogen (g/L) | 205.44 ± 132.72 | 270.50 ± 126.75 | 0.176 |
| Aspartate aminotransferase (U/L) | 195.88 ± 199.44 | 123.70 ± 221.43 | 0.332 |
| Alanine aminotransferase (U/L) | 190.32 ± 332.62 | 104.40 ± 146.80 | 0.434 |
| Lactate dehydrogenase (U/L) | 1168.79 ± 794.87 | 693.70 ± 687.51 | 0.095 |
| Albumin (g/L) | 31.70 ± 5.82 | 36.57 ± 6.65 | 0.03 |
| Total bilirubin (umol/L) | 19.11 ± 24.16 | 16.58 ± 9.36 | 0.749 |
| Activated Partial Thromboplastin Time (sec) | 56.72 ± 82.47 | 35.45 ± 10.70 | 0.424 |
| Prothrombin Time (sec) | 13.13 ± 1.98 | 12.05 ± 2.73 | 0.171 |
