# Supplementary material for: Childhood malignancy-associated hemophagocytic lymphohistiocytosis: a retrospective, single-center study of 44 patients
Source: Front Immunol. 2026 May 7;17:1801752. doi: 10.3389/fimmu.2026.1801752 (PMC13189721; doi:10.3389/fimmu.2026.1801752)
Supplement: Supplementary file 1 [file DataSheet1.zip › SupMaterial/table S1.docx]

|  | **Lymphoma**  **(n=25)** | **Acute leukemia**  **(n=12)** | **Langerhans cell histiocytosis**  **(n=7)** | **P value** |
| --- | --- | --- | --- | --- |
|  |  |  |  |  |
| Age (years) | 7.77 ± 4.36 | 4.08 ± 3.12 | 10.14 ± 3.93 | < 0.001 |
| **Gender** |  |  |  | 0.82 |
| Male | 17 | 9 | 4 |  |
| Female | 8 | 3 | 3 |  |
| **The form of HLH** |  |  |  | < 0.001 |
| Malignancy - induced HLH | 24 | 9 | 1 |  |
| Chemotherapy - induced HLH | 1 | 3 | 6 |  |
| **Manifestations** | | | | |
| Fever | 23 | 11 | 6 | 0.8 |
| Lymphadenectasis | 22 | 6 | 2 | 0.03 |
| Hepatomegaly | 20 | 10 | 7 | 0.64 |
| Splenomegaly | 18 | 9 | 5 | 1 |
| **Lab test** | | | | |
| Hemophagocytosis phenomenon in BM | 17 | 7 | 6 | 0.54 |
| EBV infection | 9 | 2 | 2 | 0.49 |
| Neutrophil (×109 /L) | 2.93 ± 5.43 | 1.42 ± 1.86 | 4.90 ± 7.05 | 0.355 |
| Hemoglobin (g/L) | 90.36 ± 18.32 | 83.92 ± 19.87 | 91.86 ± 12.08 | 0.534 |
| Platelet (×109 /L) | 82.64 ± 59.11 | 58.83 ± 74.97 | 105.57 ± 54.83 | 0.292 |
| Ferritin (ng/ml) | 5055.74 ± 8595.80 | 3532.59 ± 4340.19 | 1524.87 ± 2035.06 | 0.478 |
| Triglyceride (mmol/L) | 2.69 ± 1.61 | 2.58± 1.44 | 2.24± 0.94 | 0.78 |
| Fibrinogen (g/L) | 188.64 ± 144.85 | 240.33 ± 88.26 | 298.57 ± 126.89 | 0.127 |
| Aspartate aminotransferase (U/L) | 117 (14-570) | 200.5(226 - 864) | 41 (21 - 93) | 0.055 |
| Alanine aminotransferase (U/L) | 52(11-417) | 97.5(10-1750) | 45 (13-110) | 0.32 |
| Lactate dehydrogenase (U/L) | 1079.68 ± 736.14 | 1252.83 ± 953.97 | 664.29 ± 616.45 | 0.295 |
| Albumin (g/L) | 31.30 ± 5.80 | 34.44 ± 7.11 | 35.37 ± 5.80 | 0.184 |
| Total bilirubin (umol/L) | 15.46 ± 18.51 | 27.33 ± 29.85 | 14.47 ± 11.49 | 0.259 |
| Activated Partial Thromboplastin Time (sec) | 64.54 ± 95.34 | 36.69 ± 9.25 | 32.73 ± 9.35 | 0.426 |
| Prothrombin Time (sec) | 13.42 ± 2.21 | 12.70 ± 1.82 | 11.30 ± 2.08 | 0.068 |
