# Supplementary material for: Childhood malignancy-associated hemophagocytic lymphohistiocytosis: a retrospective, single-center study of 44 patients
Source: Front Immunol. 2026 May 7;17:1801752. doi: 10.3389/fimmu.2026.1801752 (PMC13189721; doi:10.3389/fimmu.2026.1801752)
Supplement: Supplementary file 1 [file DataSheet1.zip › SupMaterial/Table1.docx]

Table 1 Baseline M - HLH patient characteristics

| **Characteristics** | **Median (range) or N (%)** |
| --- | --- |
| Gender (male) | 30 (68.2%) |
| Age ≥ 10 year | 18 (40.9%) |
| **Lymphoma subtype** | |
| T/NK-cell lymphoma | 16 (36.4%) |
| B-cell lymphoma | 3 (6.8%) |
| Hodgkin lymphoma | 3 (6.8%) |
| Unknown lymphoma type | 3 (6.8%) |
| **AL** | |
| ALL | 7 (15.9%) |
| MDS-AML | 1 (2.3%) |
| JMML | 4 (9.1%) |
| **LCH** | 7 (15.9%) |
| **The form of HLH** | |
| Malignancy - induced HLH | 35 (79.5%) |
| Chemotherapy-induced HLH | 9 (20.5%) |
| **Clinical manifestations** | |
| Fever | 40 (91%) |
| Splenomegaly | 32 (72.7%) |
| Hepatomegaly | 37 (72.7%) |
| Lymphadenopathy | 30 (68.2%) |
| **Lab test** | |
| EBV infection | 13 (29.5%) |
| Neutrophil (×10^9^ /L) | 1.09 (0.10 - 23.14) |
| Hemoglobin (g/L) | 88.8 ± 17.8 |
| Platelet (×10^9^ /L) | 54 (8 - 289) |
| Ferritin (ng/mL) | 1424.2 (106.30 - 40023) |
| Triglyceride  (mmol/L) | 2.23 (1 - 9.67) |
| Fibrinogen (g/L) | 168 (50 - 669) |
| Aspartate aminotransferase (U/L) | 90.5 (14 - 864) |
| Alanine aminotransferase (U/L) | 53.5 (10 - 1750) |
| Lactate dehydrogenase (U/L) | 773 (169- 2933) |
| Albumin (g/L) | 32.8 ± 6.3 |
| Total bilirubin (umol/L) | 10.1 (2 - 102.6) |
| Activated Partial Thromboplastin Time (sec) | 37.05 (19 - 507) |
| Prothrombin Time (sec) | 12.8 (8.9 - 19.9) |
| Hemophagocytosis phenomenon in BM | 30 (68.2%) |

*ALL:* Acute Lymphoblastic Leukemia; *AML:* Acute Myeloid Leukemia; *MDS:* Myelodysplastic Syndrome; *LCH:* Langerhans Cell Histiocytosis; *JMML:* Juvenile Myelomonocytic Leukemia
