# Supplementary material for: Childhood malignancy-associated hemophagocytic lymphohistiocytosis: a retrospective, single-center study of 44 patients
Source: Front Immunol. 2026 May 7;17:1801752. doi: 10.3389/fimmu.2026.1801752 (PMC13189721; doi:10.3389/fimmu.2026.1801752)
Supplement: Supplementary file 1 [file DataSheet1.zip › SupMaterial/Table3.docx]

Table 3 factor analysis of risk factors for death in M-HLH

| **Factors** | **Univariate analysis** | | | **Multivariate Analysis** | | |
| --- | --- | --- | --- | --- | --- | --- |
|  | **HR** | **95%CI** | **P value** | **HR** | **95%CI** | **P value** |
| Age < 10 years | 0.363 | 0.131-1.001 | 0.05 |  |  |  |
| PLT ≥ 100×10^9^/L | 0.17 | 0.022-1.286 | 0.086 |  |  |  |
| SF ≤ 5000 ng/mL | 0.219 | 0.081-0.59 | 0.003 |  |  |  |
| AST < 80 U/L | 0.358 | 0.115-1.111 | 0.075 |  |  |  |
| LDH < 500 U/L | 0.239 | 0.054-1.054 | 0.059 |  |  |  |
| The malignancies' direct treatment | 0.506 | 0.237-1.084 | 0.08 |  |  |  |
| CR at the final follow-up | 0.036 | 0.01-0.138 | < 0.001 | 0.036 | 0.01-0.138 | <0.001 |
| CR at 4 weeks | 0.149 | 0.042-0.529 | 0.003 |  |  |  |

*AST: Aspartate aminotransferase. LDH :*Lactate dehydrogenase, *SF :*Serum ferritin. *PLT*: Platelet count.
