# Supplementary material for: Childhood malignancy-associated hemophagocytic lymphohistiocytosis: a retrospective, single-center study of 44 patients
Source: Front Immunol. 2026 May 7;17:1801752. doi: 10.3389/fimmu.2026.1801752 (PMC13189721; doi:10.3389/fimmu.2026.1801752)
Supplement: Supplementary file 2 [file DataSheet2.zip › Re-submit supmaterial/Table and Figure.docx]

Table 1 Baseline M - HLH patient characteristics

| **Characteristics** | **Median (range) or N (%)** |
| --- | --- |
| Gender (male) | 30 (68.2%) |
| Age ≥ 10 year | 18 (40.9%) |
| **Lymphoma subtype** | |
| T/NK-cell lymphoma | 16 (36.4%) |
| B-cell lymphoma | 3 (6.8%) |
| Hodgkin lymphoma | 3 (6.8%) |
| Unknown lymphoma type | 3 (6.8%) |
| **AL** | |
| ALL | 7 (15.9%) |
| MDS-AML | 1 (2.3%) |
| JMML | 4 (9.1%) |
| **LCH** | 7 (15.9%) |
| **The form of HLH** | |
| The HLH at malignancy diagnosis | 35 (79.5%) |
| The HLH after malignancy chemotherapy | 9 (20.5%) |
| **Clinical manifestations** | |
| Fever | 40 (91%) |
| Splenomegaly | 32 (72.7%) |
| Hepatomegaly | 37 (72.7%) |
| Lymphadenopathy | 30 (68.2%) |
| **Lab test** | |
| EBV infection | 13 (29.5%) |
| Neutrophil (×10^9^ /L) | 1.09 (0.10 - 23.14) |
| Hemoglobin (g/L) | 88.8 ± 17.8 |
| Platelet (×10^9^ /L) | 54 (8 - 289) |
| Ferritin (ng/mL) | 1424.2 (106.30 - 40023) |
| Triglyceride  (mmol/L) | 2.23 (1 - 9.67) |
| Fibrinogen (g/L) | 168 (50 - 669) |
| Aspartate aminotransferase (U/L) | 90.5 (14 - 864) |
| Alanine aminotransferase (U/L) | 53.5 (10 - 1750) |
| Lactate dehydrogenase (U/L) | 773 (169- 2933) |
| Albumin (g/L) | 32.8 ± 6.3 |
| Total bilirubin (umol/L) | 10.1 (2 - 102.6) |
| Activated Partial Thromboplastin Time (sec) | 37.05 (19 - 507) |
| Prothrombin Time (sec) | 12.8 (8.9 - 19.9) |
| Hemophagocytosis phenomenon in BM | 30 (68.2%) |

*ALL:* Acute Lymphoblastic Leukemia; *AML:* Acute Myeloid Leukemia; *MDS:* Myelodysplastic Syndrome; *LCH:* Langerhans Cell Histiocytosis; *JMML:* Juvenile Myelomonocytic Leukemia

Table 2 Baseline characteristics of patients with Malignancy-induced HLH and Chemotherapy-induced HLH

|  | **HLH at malignancy**  **diagnosis group** | **HLH after malignancy**  **chemotherapy group** | ***P* value** |
| --- | --- | --- | --- |
| Age (years) | 9.83(0.33-15.58) | 2.33(0.33-12.75) | 0.008 |
| Gender(Male/Female) | 23 | 7 | 1 |
| Fever (Yes) | 31 | 9 | 1 |
| Lymphadenectasis | 27 | 3 | 0.06 |
| Hepatomegaly | 29 | 8 | 0.65 |
| Splenomegaly | 26 | 6 | 0.42 |
| Hemophagocytosis phenomenon in BM | 22 | 8 | 0.46 |
| EBV infection | 12 | 1 | 0.24 |
| Neutrophil (×10^9^ /L) | 2.50 ± 4.80 | 3.97 ± 5.98 | 0.425 |
| Hemoglobin (g/L) | 87.91 ± 18.84 | 92.00 ± 14.31 | 0.53 |
| Platelet (×10^9^ /L) | 69.68 ± 55.71 | 114.20 ± 78.96 | 0.05 |
| Ferritin (ng/ml) | 4778.94 ± 7747.37 | 1722.45 ± 1701.30 | 0.226 |
| Triglyceride (mmol/L) | 2.73 ± 1.56 | 2.09 ± 0.96 | 0.225 |
| Fibrinogen (g/L) | 205.44 ± 132.72 | 270.50 ± 126.75 | 0.176 |
| Aspartate aminotransferase (U/L) | 195.88 ± 199.44 | 123.70 ± 221.43 | 0.332 |
| Alanine aminotransferase (U/L) | 190.32 ± 332.62 | 104.40 ± 146.80 | 0.434 |
| Lactate dehydrogenase (U/L) | 1168.79 ± 794.87 | 693.70 ± 687.51 | 0.095 |
| Albumin (g/L) | 31.70 ± 5.82 | 36.57 ± 6.65 | 0.03 |
| Total bilirubin (umol/L) | 19.11 ± 24.16 | 16.58 ± 9.36 | 0.749 |
| Activated Partial Thromboplastin Time (sec) | 56.72 ± 82.47 | 35.45 ± 10.70 | 0.424 |
| Prothrombin Time (sec) | 13.13 ± 1.98 | 12.05 ± 2.73 | 0.171 |

Table 3 factor analysis of risk factors for death in M-HLH

| **Factors** | **Univariate analysis** | | | **Multivariate Analysis** | | |
| --- | --- | --- | --- | --- | --- | --- |
|  | **HR** | **95%CI** | **P value** | **HR** | **95%CI** | **P value** |
| Age < 10 years | 0.363 | 0.131-1.001 | 0.05 |  |  |  |
| PLT ≥ 100×10^9^/L | 0.17 | 0.022-1.286 | 0.086 |  |  |  |
| SF ≤ 5000 ng/mL | 0.219 | 0.081-0.59 | 0.003 |  |  |  |
| AST < 80 U/L | 0.358 | 0.115-1.111 | 0.075 |  |  |  |
| LDH < 500 U/L | 0.239 | 0.054-1.054 | 0.059 |  |  |  |
| The malignancies' direct treatment | 0.506 | 0.237-1.084 | 0.08 |  |  |  |
| CR at the final follow-up | 0.036 | 0.01-0.138 | < 0.001 | 0.036 | 0.01-0.138 | <0.001 |
| CR at 4 weeks | 0.149 | 0.042-0.529 | 0.003 |  |  |  |

*AST: Aspartate aminotransferase. LDH :*Lactate dehydrogenase, *SF :*Serum ferritin. *PLT*: Platelet count.


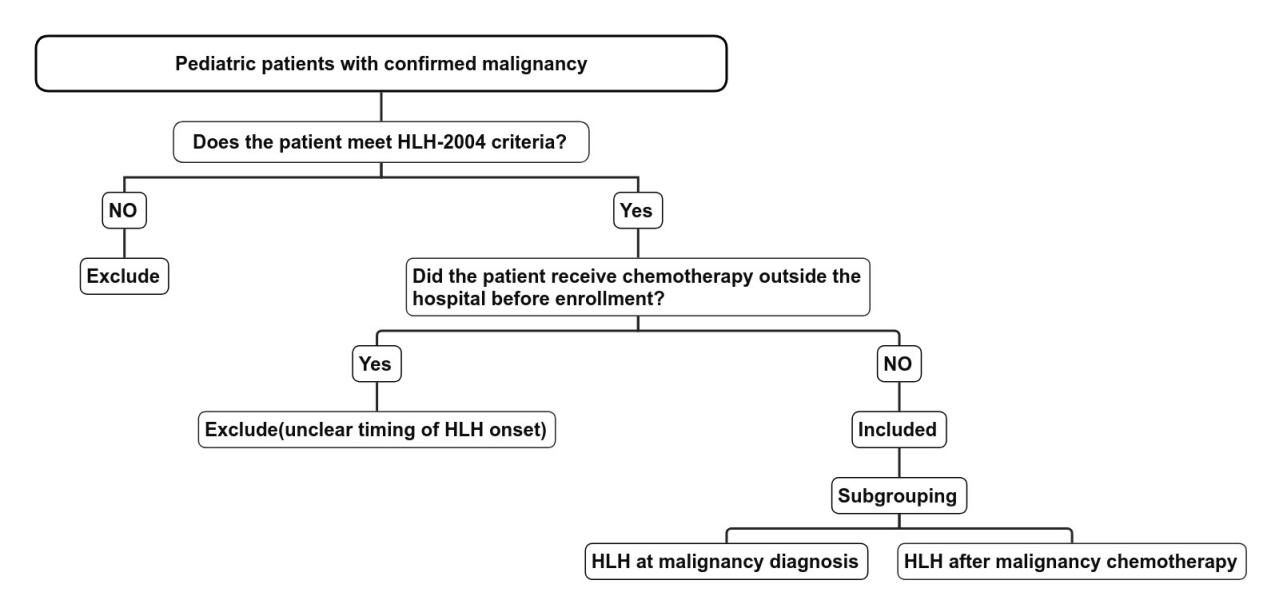


Figure 1. Flowchart of inclusion and exclusion criteria for M-HLH.


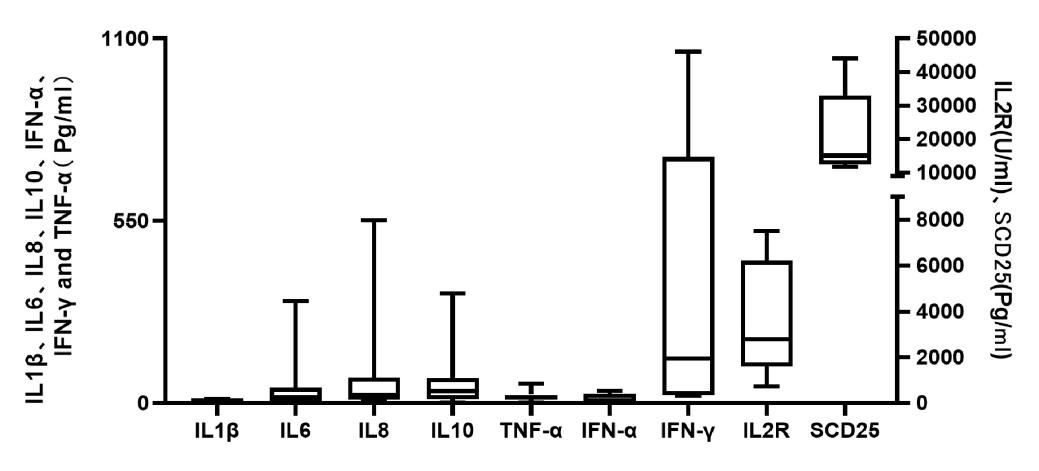


Figure 2.Laboratory results of cytokines and sCD25 in M-HLH


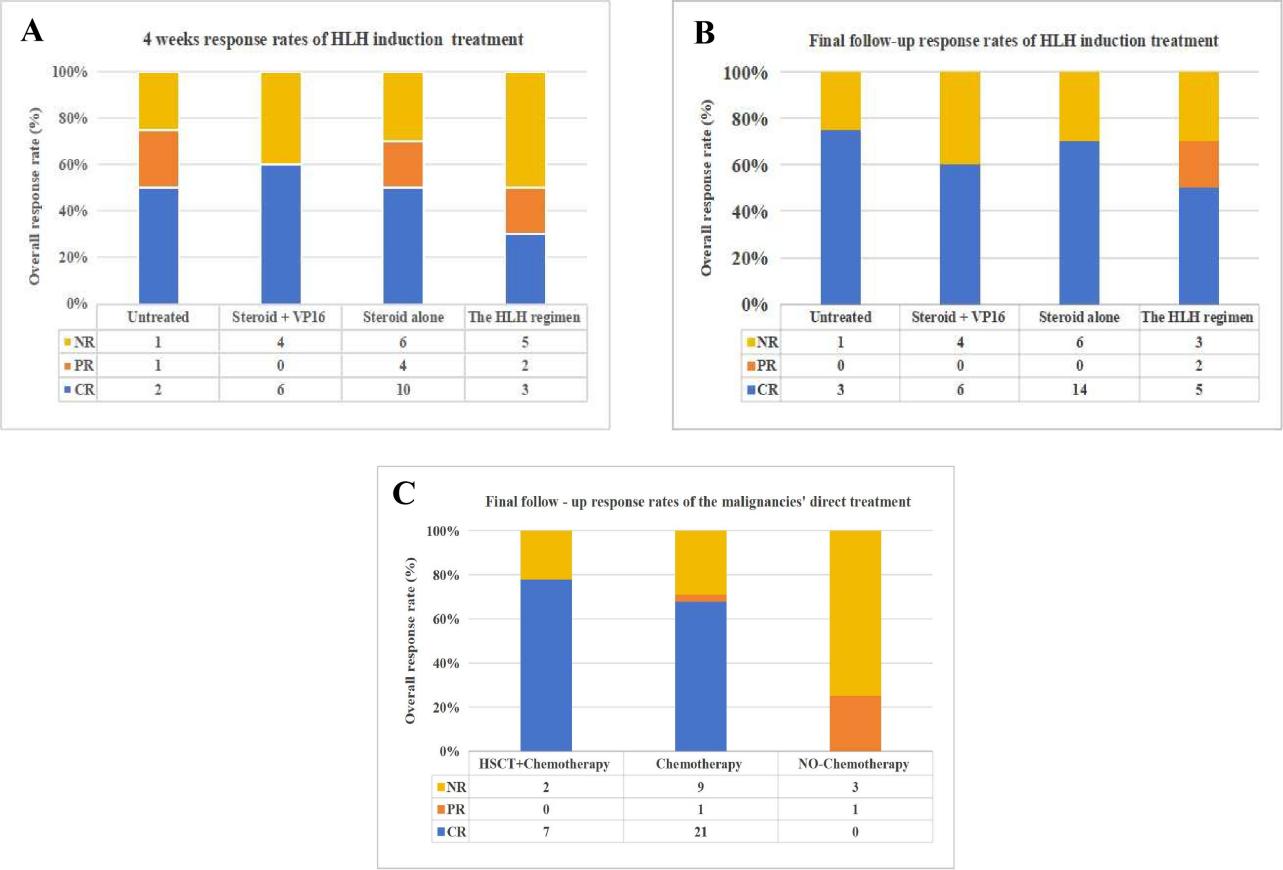


Figure 3.The response rates for HLH induction and the malignancies' direct treatment


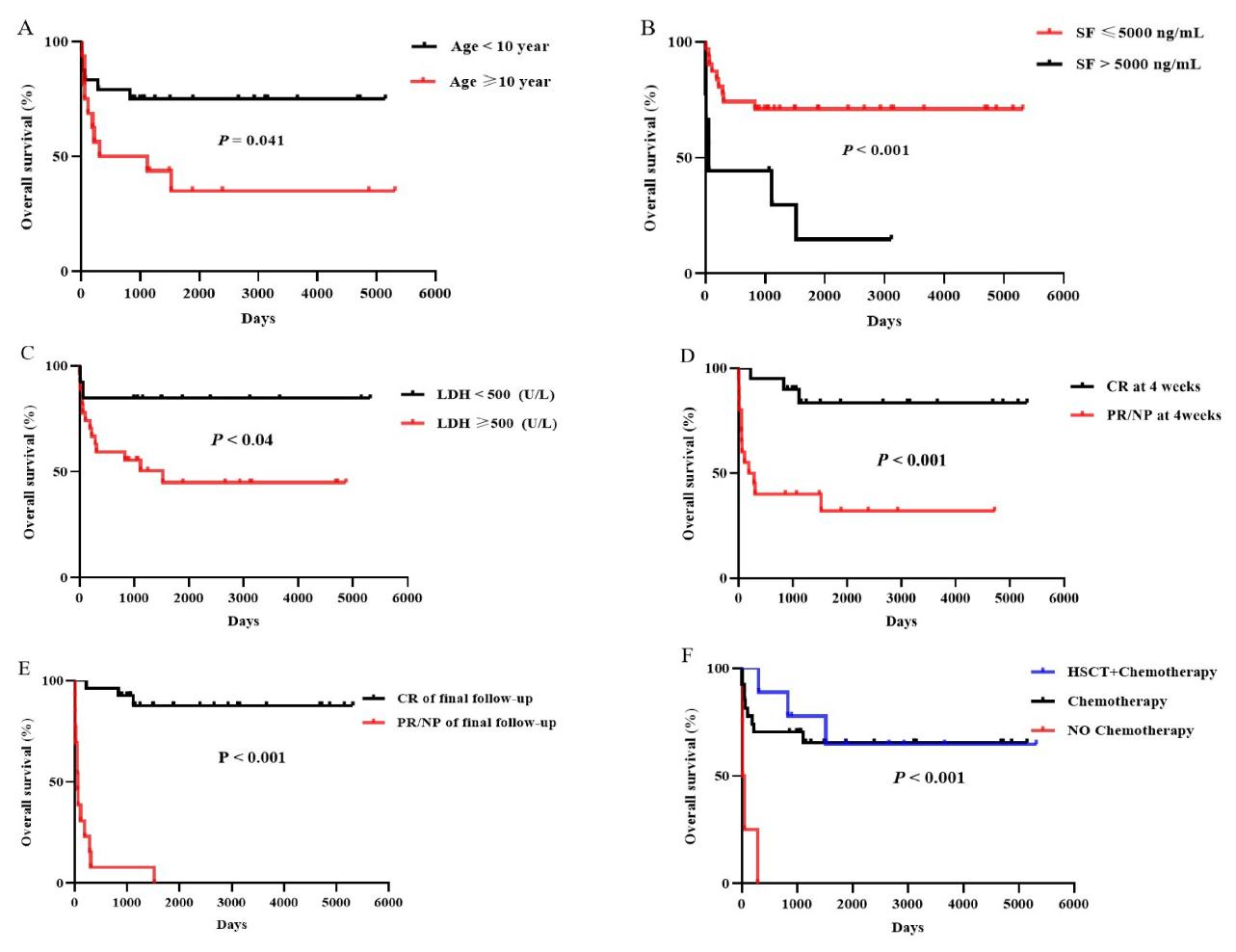
Figure 4. Overall survival of patients with M-HLH. Comparisons of overall survival based on (A) age, (B) serum ferritin (SF) level, (C) lactate dehydrogenase (LDH) level, (D) Achievement of complete response (CR) to HLH at 4 weeks; (E) achievement of CR to HLH at final follow-up; (F) Direct treatment of underlying malignancies. Abbreviations: HLH: hemophagocytic lymphohistiocytosis; CR: complete response; NR: no response; PR: partial response; HSCT: hematopoietic stem cell transplantation; LDH:lactate dehydrogenase; SF: serum ferritin.


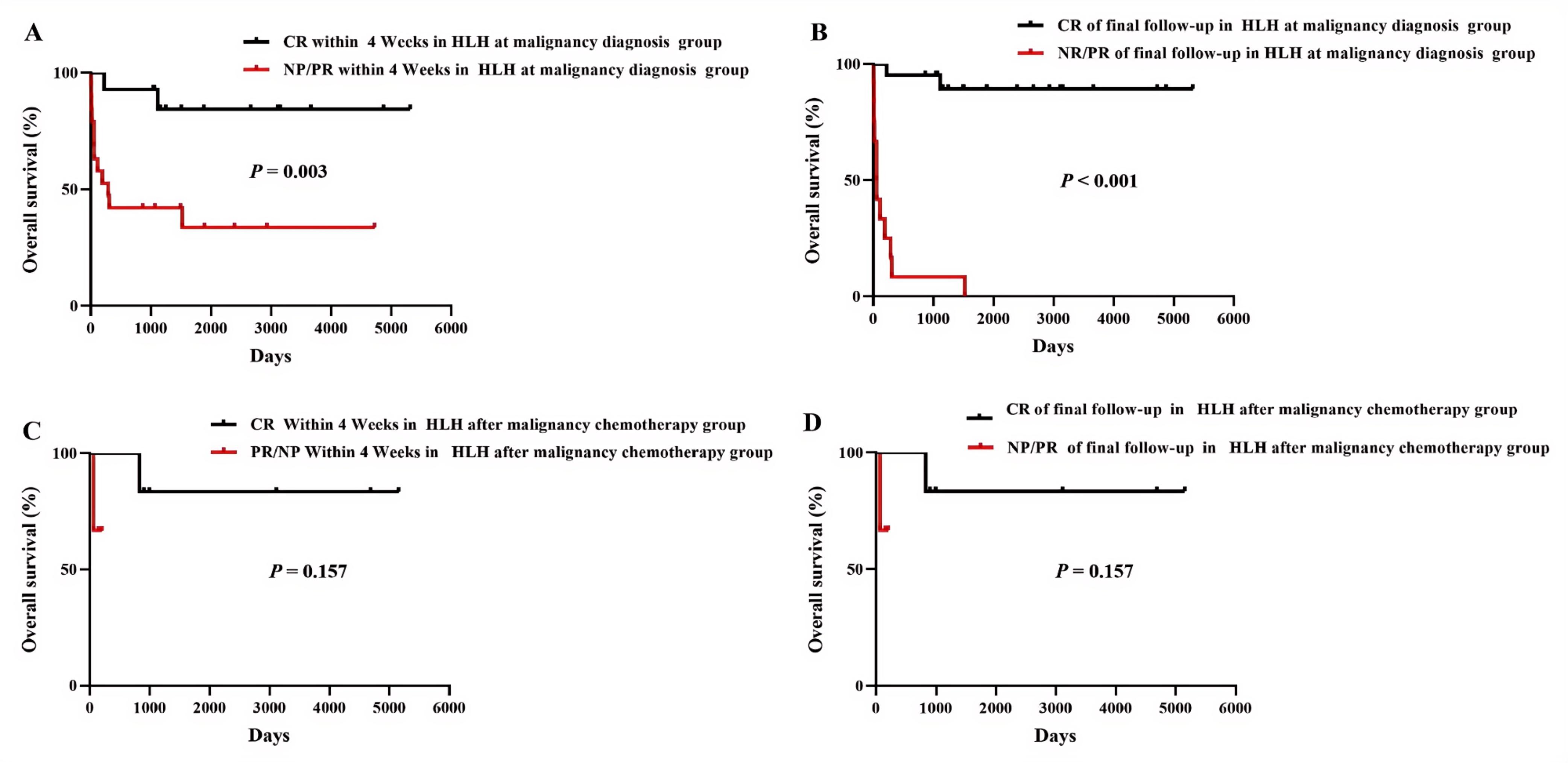


Figure 5.Overall survival of patients with CR of HLH in the HLH at malignancy diagnosis group and the HLH after malignancy chemotherapy group
