# Supplementary material for: Childhood malignancy-associated hemophagocytic lymphohistiocytosis: a retrospective, single-center study of 44 patients
Source: Front Immunol. 2026 May 7;17:1801752. doi: 10.3389/fimmu.2026.1801752 (PMC13189721; doi:10.3389/fimmu.2026.1801752)
Supplement: Supplementary file 2 [file DataSheet2.zip › Re-submit supmaterial/Reviewer 1.docx]

****Response to Reviewers****

****Manuscript ID: 1801752****

****Title:**Childhood Malignancy-Associated Hemophagocytic Lymphohistiocytosis: A Retrospective, Single-Center Study of 44 Patients**

****Journal: *Frontiers in Immunology*****

****Dear Editor and Reviewers,****

**Thank you very much for your letter and the valuable comments regarding our manuscript (Manuscript ID: 1801752). We appreciate the positive feedback on the significance of our study in addressing the gap in pediatric malignancy‑associated hemophagocytic lymphohistiocytosis (M‑HLH). We have carefully considered all the comments and have revised the manuscript accordingly. A point‑by‑point response to the reviewers’ comments is provided below. We hope that the revised version is now acceptable for publication in Frontiers in Immunology.**

**Major Comments**

**Comment 1: The study design and patient selection (page 2, lines 15-16; page 5, lines 60-65) are clearly described, but the exclusion of patients who received chemotherapy prior to admission should be justified to clarify potential selection bias.**

Response: Thank you very much for your valuable comment. Regarding the description of the inclusion and exclusion criteria, we acknowledge that the original text was not clearly expressed. We have revised it accordingly in the main text (see page 3, lines 95–104). The reason for excluding patients who had received chemotherapy outside our hospital prior to admission is that the specific chemotherapy regimens were unknown, and it was difficult to determine whether HLH occurred before or after tumor chemotherapy. In this study, we divided enrolled patients into two groups for comparative analysis: “HLH occurring at the time of tumor diagnosis” and “HLH occurring after tumor chemotherapy.” Patients who had received chemotherapy at outside hospitals could not be accurately assigned to either group and were therefore excluded. In addition, these patients received only anti-infective therapy at our institution and did not continue tumor-related chemotherapy. Therefore, we believe that this exclusion criterion does not introduce selection bias. Furthermore, to more clearly present the patient screening process, we have added a flowchart in the revised manuscript (Figure 1).

**Comment 2:The statistical analysis methods (page 6, lines 84-90) are appropriate, but the manuscript would benefit from specifying how missing data were handled and whether any sensitivity analyses were performed.**

Response: Thank you for this valuable suggestion. Regarding the handling of missing data, key laboratory parameters (including complete blood count, liver and renal function, electrolytes, coagulation function, ferritin, EBV infection status, etc.) and HLH response assessment indicators were complete in this study. For patients with no documented death outcome in our hospital’s electronic medical records, we supplemented this information through telephone follow-up. For variables with partial missing data, such as soluble CD25, cytokines, NK cell activity, and genetic testing results, we analyzed them descriptively by treating “missing” as a separate category and did not include them in the regression models for prognostic factors or survival analysis to control for potential bias. Since the main prognostic analyses only included key variables with complete data and the missing variables were not involved in the primary outcome inference, we consider their impact on the study conclusions to be minimal; therefore, no sensitivity analysis was performed. We have supplemented the corresponding description in the “Statistical Analysis” section on page 3 of the main text

**Comment 3:The results (page 8, lines 92-152) are well-structured, though the reporting of response rates and survival outcomes could be enhanced by including confidence intervals for key estimates (e.g., ORR, OS rates).**

Thank you for this valuable suggestion. We have added 95% confidence intervals for key estimates in the Results section and updated the overall survival rates using the Kaplan‑Meier method. The overall response rate (ORR) was 63.6% (95% CI: 48.6%–78.0%) at 4 weeks and 68.2% (95% CI: 53.4%–83.0%) at the final follow-up. The overall survival (OS) rates were 79.0% (95% CI: 64.2%–88.2%) at 6 months, 69.0% (95% CI: 53.0%–80.5%) at 1 year, and 67.0% (95% CI: 51.3%–78.7%) at 2 years. These revisions have been incorporated on page 6.

**Comment 4: The discussion (page 13, lines 163-227) effectively contextualizes findings, but the limitations section should more explicitly address the retrospective design's impact on data completeness and potential confounding factors.**

Response:Thank you for this valuable suggestion. In response, we have substantially expanded and revised the limitations section to more explicitly address the impact of the retrospective design on data completeness and to detail potential confounding factors. Specifically, we now describe the following: the restriction in analytical scope due to missing data for key immunological parameters (e.g., soluble CD25, cytokines, NK cell activity) and genetic testing; the inability to perform robust multivariate analyses to adjust for confounders (such as malignancy subtype, timing of HLH onset, and treatment intensity) due to the limited sample size; unmeasured confounding arising from the heterogeneity of underlying malignancies and treatment protocols; and potential information bias introduced by variability in follow-up duration. These revisions have been incorporated in the “Limitations and Future Directions” section on page 9.

Minor Comments

**Comment 1: Page 2, line 13: The abstract purpose could be more precise by stating the study's retrospective nature upfront.**

Response:Thank you for this suggestion. We have revised the abstract to clearly state the retrospective nature of the study upfront. The Purpose section now reads: “This retrospective study aimed to investigate the clinical characteristics, management, and prognosis of pediatric malignancy-associated hemophagocytic lymphohistiocytosis (M-HLH).” The change has been made on page1, line 13.

**Comment 2:Page 9, lines 104-108: The reporting of genetic testing results is brief; consider elaborating on the clinical relevance of identified variants.**

Response:Thank you for this valuable suggestion. We have elaborated on the reporting of genetic testing results on page 5, lines 152–159. Genetic testing was performed on 10 patients, and pathogenic variants in HLH-associated genes (LYST, UNC13D, and XIAP) were identified in three cases. The details are as follows:

One patient with LCH carried an exon 2 deletion mutation in the XIAP gene; this patient developed HLH during chemotherapy and was EBV-negative.

One patient with B-cell lymphoma carried missense mutations in exon 19 of the UNC13D gene and exon 6 of the LYST gene; this patient had HLH onset at the time of tumor diagnosis and was EBV-positive.

One patient with T-cell lymphoma carried missense mutations in exon 27 of the UNC13D gene, as well as missense mutations in exon 20 and exon 31; this patient also had HLH onset at the time of tumor diagnosis and was EBV-positive.

In addition, we have further discussed the clinical relevance of these findings—specifically the complex interplay between genetic susceptibility, malignancy, chemotherapy, and infection—in the Discussion section (page 7, lines 241–258).

**Comment 3:Page 12, line 227: The abbreviations list is helpful but could be moved to a supplementary section for readability.**

Response:Following the reviewer’s suggestion, we have removed the abbreviations list from the main text to improve readability. The list is now provided as a supplementary file **(Supplementary Table S2).** The main text has been updated accordingly, with the first use of each abbreviation defined in the text.

**Comment 4:Page 14, lines 244-245: Funding sources are clearly stated, but the role of funders in study design and reporting should be clarified.**

Response:Thank you for this important point. We have clarified the role of the funders on page 10, lines 337–340. The funding sources (National Natural Science Foundation of China, Grant No. 81901521; Natural Science Foundation of Sichuan Province, China, Grant No. 2022NSFSC1501) provided financial support for publication fees and had no involvement in the study design, data collection, analysis, interpretation, or manuscript preparation.

**Suggestions**

**Suggestion1:Consider adding a flow diagram summarizing patient inclusion/exclusion.**

Response: Thank you for this suggestion. We have added a flow diagram summarizing patient inclusion and exclusion (Figure 1) to improve methodological transparency.

**Suggestion 2:Provide more detail on follow-up duration and loss to follow-up.**

Response:Thank you for this suggestion. We have now provided more detailed information on follow-up duration and loss to follow-up in the manuscript. Specifically, we have clarified the definition of overall survival, specified the follow-up cutoff date (December 2024), reported the median follow-up duration, and noted that loss to follow-up occurred in 4 patients (9.1%).

**Suggestion 3:Clarify the rationale for classifying LCH as a malignancy in this context.**

Response:Thank you for this important point. We have clarified the rationale for classifying Langerhans cell histiocytosis (LCH) as a malignancy in this context in the manuscript. LCH is a heterogeneous disorder; its clinical behavior ranges from self‑limited to aggressive, with multisystem involvement—particularly with risk organ involvement—associated with high recurrence rates and poor prognosis. In our cohort, all patients with LCH had multisystem disease with risk organ involvement, and they were treated with chemotherapy analogous to that used for malignant tumors. Therefore, we classified LCH as a malignancy in this study. This clarification has been added on page 3, lines 101–104.
