# Supplementary material for: Childhood malignancy-associated hemophagocytic lymphohistiocytosis: a retrospective, single-center study of 44 patients
Source: Front Immunol. 2026 May 7;17:1801752. doi: 10.3389/fimmu.2026.1801752 (PMC13189721; doi:10.3389/fimmu.2026.1801752)
Supplement: Supplementary file 2 [file DataSheet2.zip › Re-submit supmaterial/Reviewer 2.docx]

****Response to Reviewers****

****Manuscript ID: 1801752****

****Title:**Childhood Malignancy-Associated Hemophagocytic Lymphohistiocytosis: A Retrospective, Single-Center Study of 44 Patients**

****Journal: *Frontiers in Immunology*****

****Dear Editor and Reviewers,****

**Thank you very much for your letter and the valuable comments regarding our manuscript (Manuscript ID: 1801752). We appreciate the positive feedback on the significance of our study in addressing the gap in pediatric malignancy‑associated hemophagocytic lymphohistiocytosis (M‑HLH). We have carefully considered all the comments and have revised the manuscript accordingly. A point‑by‑point response to the reviewers’ comments is provided below. We hope that the revised version is now acceptable for publication in Frontiers in Immunology.**

1. **HLH is fundamentally a hyperinflammatory syndrome driven by excessive activation of T cells and macrophages, characterized by a cytokine storm involving IFN-γ, IL-6, IL-10, IL-18, and CXCL9. The authors are encouraged to strengthen the immunopathological interpretation of the observed prognostic differences. If available, retrospective analysis of serum cytokines (e.g., IFN-γ, IL-10, IL-18, CXCL9) at diagnosis or during treatment would significantly enhance the mechanistic insight. If these data are unavailable, this limitation should be clearly acknowledged and future prospective studies incorporating cytokine profiling should be suggested. The discussion should also elaborate on the potential clinical implications, particularly whether HLH arising in different immune contexts may require different therapeutic priorities.**

Response: Thank you for this insightful comment. In response, we have incorporated available cytokine data and strengthened the immunopathological interpretation in the Discussion section. A total of 17 patients underwent cytokine testing, including IL‑1β, IL‑2R, IL‑6, IL‑8, IL‑10, TNF‑α, IFN‑α, and IFN‑γ. Marked elevations were observed in IL‑2R, IFN‑γ, TNF‑α, IL‑6, and IL‑10 (Figure 1). In the Discussion, we have elaborated on the relationship between cytokine profiles and M‑HLH, with particular attention to the potential role of IL‑2R as an alternative to sCD25 for assessing activation status in M‑HLH, given that sCD25 is not routinely available in many clinical settings. We have also acknowledged the limitation that only two patients with chemotherapy‑induced HLH had cytokine data available, precluding a comparative analysis between the two subgroups. These revisions have been incorporated on page 5, lines 152–163 and page 8, lines 259–270 .

2. **The definition of malignancy-associated HLH is heterogeneous in the literature. The distinction between malignancy-induced and chemotherapy-induced HLH is a strength of this study but requires clearer definition in the Methods section to ensure reproducibility. A concise operational definition should be explicitly stated in Section**

Response: Thank you for this important comment. We apologize for the lack of clarity in the initial definition. In response, we have revised the description in the Methods section to explicitly define the two subgroups. Specifically, “malignancy‑induced HLH” has been modified to “**HLH at malignancy diagnosis**,” and “**HLH after malignancy chemotherapy**” has been modified to “HLH occurring after tumor chemotherapy.” These operational definitions have been clearly stated to ensure reproducibility. The corresponding revisions have been made throughout the manuscript.

3. **Three HLH-associated gene variants (LYST, UNC13D, XIAP) were identified in the subset of patients undergoing genetic testing. Although limited in number, these findings warrant deeper discussion. The authors should briefly describe the immunological functions of these genes and discuss how genetic susceptibility may interact with triggers such as malignancy, chemotherapy, or EBV infection to precipitate HLH. Recent literature on the potential impact of these variants on immune surveillance or treatment tolerance may also be considered**

Response: Thank you for this insightful comment. We have now expanded the discussion regarding the three HLH-associated gene variants (LYST, UNC13D, and XIAP). The revised discussion addresses the immunological functions of these genes and explores how genetic susceptibility may interact with triggers such as malignancy, chemotherapy, or EBV infection to precipitate HLH, with reference to recent literature. These additions can be found on page 7, lines 241–258.
